# Silencing GTSE-1 expression inhibits proliferation and invasion of hepatocellular carcinoma cells

**DOI:** 10.1007/s10565-016-9327-z

**Published:** 2016-05-30

**Authors:** Lei Guo, Shumin Zhang, Bo Zhang, Wanyong Chen, Xiaoqiang Li, Wentao Zhang, Chenhao Zhou, Jubo Zhang, Ning Ren, Qinghai Ye

**Affiliations:** Liver Cancer Institute and Zhongshan Hospital, Fudan University, Shanghai, 200032 China; Key Laboratory of Carcinogenesis and Cancer Invasion, Fundan University, Ministry of Education, Shanghai, 200032 China; Department of Radiation Oncology, Zhongshan Hospital, Fudan University, Shanghai, 200032 China; Department of Liver Surgery, Zhongshan Hospital, Fudan University, No.180 Fenglin Road, Shanghai, 200032 China

**Keywords:** HCC, GTSE1, Proliferation, Invasion, Prognosis

## Abstract

G2 and S phase-expressed-1 (GTSE1) was recently reported to upregulate in several types of human cancer, based on negatively regulate p53 expression. However, its expression and functional roles in hepatocellular carcinoma (HCC) remain unknown. In this study, GTSE1 was observed to be highly expressed in HCC specimens and cell lines both at messenger RNA (mRNA) and protein levels. Furthermore, high GTSE1 expression was positively associated with tumor size, venous invasion, advanced tumor stage, and short overall survival. Moreover, we generated stable GTSE1 knockdown HCC cell lines to explore the effects of GTSE1 silencing on the growth and invasion of HCC in vitro. In determining the pathway through which GTSE1 regulated cell proliferation and invasion, GTSE1 silencing was found to inhibit AKT phosphorylation and downregulated cell cycle-related protein. In addition, GTSE1 downregulation decreased the growth of xenografts. In conclusion, these results indicated for the first time that overexpression of GTSE1 was involved in the progress of HCC, enhancing proliferation and promoting cell invasion in HCC cells.

**Electronic supplementary material:**

The online version of this article (doi:10.1007/s10565-016-9327-z) contains supplementary material, which is available to authorized users.

## Introduction

Hepatocellular carcinoma (HCC) is one of the most malignant cancers all over the world, with the third cause of cancer mortality (Poon 2011; Liu et al. 2015). A number of risk factors have been shown to drive this process, including hepatitis virus infections, non-alcoholic fatty liver diseases, and alcoholic liver diseases (Cavazza et al. 2009). Although HCC diagnosed at early stage is curable through surgical resection, patients are often diagnosed with HCC at advanced stage. Molecular biological study of HCC is thus required to develop novel therapeutic approaches.

G2 and S phase-expressed-1 (GTSE1), located in chromosome 22q13.2-q13.3, specifically expressed during S and G2 phases of the cell cycle (Utrera et al. 1998; Monte et al. 2000). GTSE1 has been previously interacted with p53 and relocalized p53 to the cytoplasm to undergo degradation (Monte et al. 2004). Further study indicated that overexpression of GTSE1 was frequently detected in human cancers. It has been shown that amplification of GTSE1 gene has been found in lung cancer, and high GTSE1 expression was associated with the histological types (Tian et al. 2011). Significantly increased GTSE1 expression has also been demonstrated in myeloma cells after cisplatin treatment (Spanswick et al. 2012). It has also been reported that upregulation of GTSE1 in gastric cancer contributed to inhibited cisplatin sensitivity via p53 apoptotic signaling (Subhash et al. 2015). However, the role of GTSE1 in the development of HCC in vitro and in vivo has not yet been elucidated.

In the current study, we show that GTSE1 was overexpressed and correlated with tumor invasion and worse outcome in HCC. Downregulation of GTSE1 inhibited the growth of HCC cells via the inactivation of PI3K/AKT pathway and its downstream apoptosis-related protein.

## Materials and methods

### Patients and samples

The 76 pairs of HCC specimens and paired non-cancerous specimens were obtained from surgical resections at the Liver Cancer Institute, Zhongshan Hospital, Fudan University between May 2006 and December 2006. No patients have received systemic or local treatment before operation. Tumor stage was determined according to the sixth edition of the American Joint Committee on Cancer tumor-node-metastasis (TNM) staging system. Informed consent was obtained from all patients. The HCC tissues enrolled were approved by the Clinical Research Ethics Board of Zhongshan Hospital affiliated to Fudan University and were performed in accordance with the Declaration of Helsinki guidelines.

### Cell lines

The HCC cell lines (SMMC-7721, Huh7, MHCC97H, and MHCCLM3), a normal hepatocyte cell line (L02), and the human embryonic kidney 293T cells were conserved and maintained in Dulbecco’s modified Eagle’s medium (DMEM) (HyClone, Logan, UT, USA) supplemented with 10 % fetal bovine serum (Invitrogen, Carlsbad, CA, USA), 100 units/ml penicillin, and 100 μg/ml streptomycin. All cells were cultured at 37 °C humidified incubator under an atmosphere of 5 % CO_2_ in air.

### RNA extraction and real-time quantitative reverse-transcription polymerase chain reaction

Total RNA was extracted from HCC tissues and cells using Trizol Reagent (Invitrogen) following the manufacturer’s instructions. One microgram total RNA was converted to first-strand cDNA using the PrimeScript RT reagent kit (TaKaRa, Japan). Real-time quantitative reverse-transcription polymerase chain reaction (qRT-PCR) was performed with the Realtime PCR Master Mix (Toyobo, Osaka, Japan) according to the manufacturers’ instructions by an ABI 7900HT real-time PCR system (Applied Biosystems, Foster City, CA, USA), following temperature profiles: 1 cycle at 95 °C (2 min), 40 cycles of denaturation at 95 °C (30 s), and annealing at 60 °C (1 min). The primer sequences used were as follows: 5′-CCACCGGGATGTTCTCCCT-3′ (forward primer) and 5′-TTCAGCCCCAACTTGTTTGGA-3′ (reward primer) for GTSE1; 5′-ACCCAGAAGACTGTGGATGG-3′ (forward primer) and 5′-CAGTGAGCTTCCCGTTCAG-3′ (reward primer) for GAPDH. Then, the 2 − ∆∆CT method was used to determine the relative gene expression levels, and each experiment was repeated at least three times.

GAPDH was used as an internal control.

### Plasmids and transfection

Short hairpin RNA (shRNA) against the human GTSE1 was designed and synthesized by the Shanghai Invitrogen, China. The optimal sequence of shRNA targeting GTSE1 (5′-CCGGGCCTACTCCTACAAATCAATTCTCGAGAATTGATTTGTAGGAGTAGGCTTTTTG-3′ (forward primer) and 5′-AATTCAAAAAGCCTACTCCTACAAATCAATTCTCGAGAATTGATTTGTAGGAGTAGGC3′ (reward primer) was then cloned into the lentiviral plasmid pLKO.1-TRC Cloning Vector. Lentivirus productions were produced as described previously (Dong et al. 2016). To generate stable shRNA knockdown cells, targeted HCC cells were added with viral supernatant and 8 μg/ml polybrene (Sigma-Aldrich, St Louis, MO, USA) for 24 h. Then the cells were treated with 2 μg/ml puromycin (Sigma) for 48 h.

### Proliferation assays

Cell viability was assessed by CCK-8 (Dojindo, Kumamoto, Japan) assay. Cells (4 × 10^3^ /well) were seeded in 96-well plate. Ten microliters CCK-8 was added to each well at 72 and 96 h. Absorbance was measured at 450 nm after incubating for another 2 h at 37°.

#### Colony formation assay

Cells were seeded into six-well plates at a density of 800 per well and incubated at 37 °C and at an atmosphere of 5 % CO_2_ for 14 days. Cells were fixed with methanol at −20 °C for 5 min and stained with 0.5 % crystal violet for 30 min. Only clearly visible colonies (more than 50 cells) were counted under a light microscope.

### Flow cytometric analysis of cell cycle analysis and cell apoptosis

For the cell cycle analysis, cells were harvested, washed with phosphate-buffered saline, and then fixed in 70 % ice-cold ethanol at 4 °C overnight. Cells were treated with RNase (final concentration 0.2 mg/ml) and stained with propidium iodide (final concentration 10 μg/ml) in the dark, following detection with a FACSCalibur Cytofluorimeter (Becton-Dickinson) as previously described (Wong et al. 2014).

For the cell apoptosis analysis, cells were harvested and stained with the Annexin V-FITC Apoptosis Detection Kit (Sigma-Aldrich) following the manufacturer’s protocol. Apoptosis was measured and analyzed as previously described (Zhu et al. 2016). All experiments were performed at least three times.

### Migration and invasion assay

Boyden chamber assay with or without coated Matrigel (BD Biosciences, Sparks, MD, USA) was used to perform the cell migration or invasion analysis, respectively. The cells (4 × 10^5^ /well) were suspended in 200 μl of serum-free medium and seeded into the upper chamber of the 8-μm pore inserts (Corning Inc., NY, USA). Six hundred-microliter medium containing 10 % fetal bovine serums was added to the lower chamber as a chemoattractant. Following 24-h incubation for migration and 48-h incubation for invasion at 37 °C, the cells on the upper surface of the filter were scraped off with a cotton swab, and the migrated or invaded cells on the lower surface were fixed and stained with 1 % crystal violet for 15 min. The number of migrated or invaded cells per field was determined by calculating six random fields. All experiments were performed in triplicate wells and repeated three times.

### Immunohistochemistry

Immunohistochemical staining was performed as previously described with a Dako Dako REAL™ Detection System (Zhu et al. 2013). In brief, 4-μm formalin-fixed paraffin sections were cut. After the antigen retrieval in citrate buffer, sections were blocked with 5 % normal goat serum at room temperature and stained with antibodies against GTSE1 (Proteintech Group, Inc., Chicago, IL, USA), followed by incubation with goat anti-Rabbit IgG-biotinylated secondary antibodies (Dakopatts, Glostrup, Denmark), and visualized by standard avidin-biotinylated peroxidase complex method. Staining intensity was evaluated by two investigators who were unaware of clinicopathological features of the patients. Dark brown staining was defined as positive, and no staining was defined as negative. The percentage of positive cells and the intensity of immunostaining were used to produce a weighted score for each case.

### Western blotting analysis

Whole-cell extracts were prepared as described previously (Liu et al. 2013). In brief, equivalent amounts of protein (50 μg) were separated by sodium dodecyl-sulfate-polyacrylamide gel electrophoresis (10 %) and transferred to nitrocellulose membrane (Bio-Rad, Hercules, CA). The membranes were subsequently incubated with the following primary antibodies: GTSE1 (Proteintech Group, Inc), AKT, phospho-AKT (Ser473), and cyclin B1 from CST (Cell Signaling Technology, Beverly, MA, USA), BCL-2 and Bax from Abcam (AbCam, Cambridge, MA, USA), and GAPDH (CST) was used as a loading control. The blots were visualized with a horseradish peroxidase-conjugated secondary antibody (Kangchen, Shanghai, China) and an enhanced chemiluminescence kit (Millipore, Bedford, MA).

### In vivo experimental tumorigenesis assay

All of the animal care and experimental procedures were performed in accordance with the Laboratory Animal Care Guidelines and approved by the Animal Care and Use Committee of Zhongshan Hospital, Fudan University.

Four-week-old male BALB/c nude mice were purchased from the Shanghai Experimental Animal Center and subcutaneously injected with 1 × 10^7^ LM3 cells transfected with negative control (SCR) or GTSE1-SH. The tumor dimensions were measured with vernier calipers every 1 week. Mice were sacrificed 5 weeks after the subcutaneous injection, following the xenografts weights were immediately measured.

### Statistical analysis

All experiments were performed in triplicate in three independent experiments. Data were expressed as means ± standard deviation (SD). Statistical analyses were performed using SPSS 16.0 (IBM, Armonk, NY, USA). Pearson’s chi-square test was performed to determine the relationship between GTSE1 expression and clinical parameters. Kaplan-Meier method was used to assess survival curves. A paired *t* test (two tails) was used for statistical analyses between two groups.

## Results

### GTSE1 is aberrantly overexpressed in HCC cell lines and cancerous tissues

**Fig. 1:**
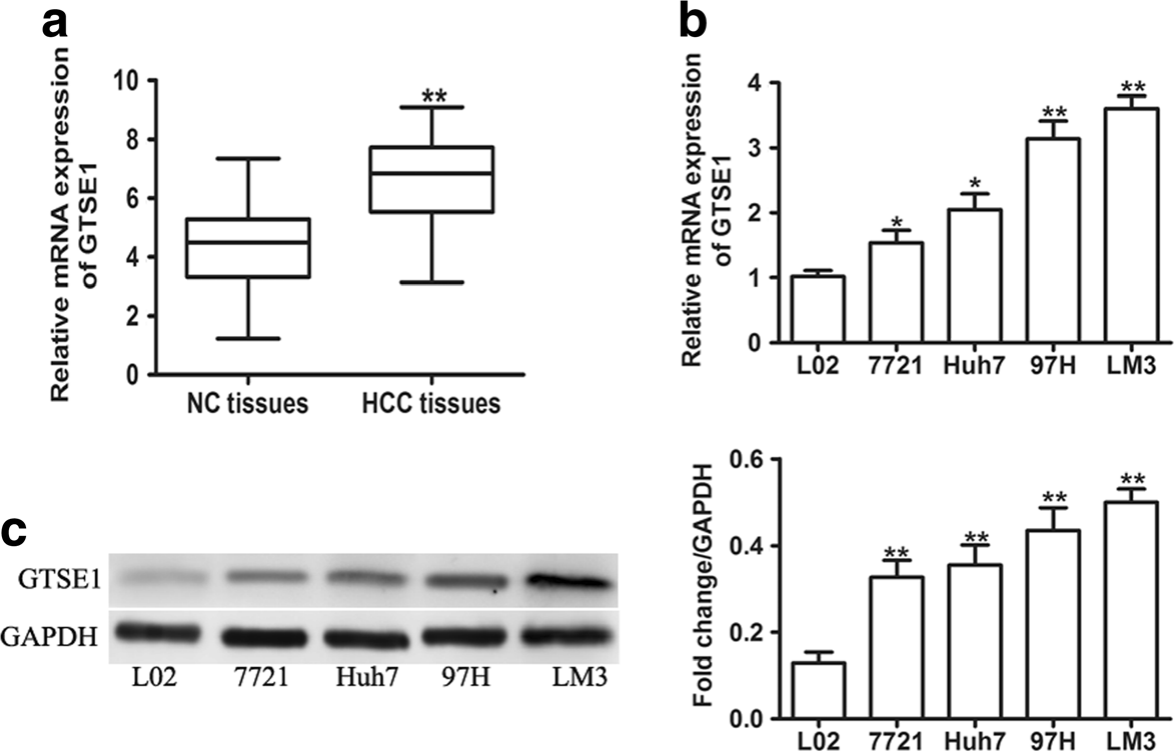
Upregulation of GTSE1 in HCC. **a** qRT-PCR analysis of mRNA levels of GTSE1 in 76 paired of HCC tissues and adjacent non-cancerous tissues (*NC tissues*). **b** The mRNA level of GTSE1 was quantified in four HCC cells and a non-malignant liver cell (*L02*). **c** The protein level of GTSE1 was determined in four HCC cells and a non-malignant liver cell (*LO2*) by western blot assays. GAPDH was used as an internal control. *P < 0.05, **P < 0.01

To investigate the expression of GTSE1 in HCC tumor samples, qRT-PCR was utilized to detect the messenger RNA (mRNA) levels of GTSE1 in HCC tumor samples and corresponding adjacent non-cancerous tissues. As shown in Fig. 1a, GTSE1 expression was significantly higher in 76 paired HCC tissues compared with paraneoplastic non-cancerous tissues. Furthermore, we measured the mRNA levels of GTSE1 in HCC cells. Interestingly, we found that GTSE1 expression was remarkably higher in HCC cells compared with non-malignant liver cells (L02) (Fig. 1b). Consistently, GTSE1 protein expression was increased in HCC cells compared with LO2 as detected by western blot, especial in 97H and LM3 (Fig. 1c). Hence, our data suggested that GTSE1 expression is upregulated in HCC.

### High GTSE1 expression is associated with tumor size, venous invasion, and advanced tumor stage and predicts poor prognosis

**Fig. 2:**
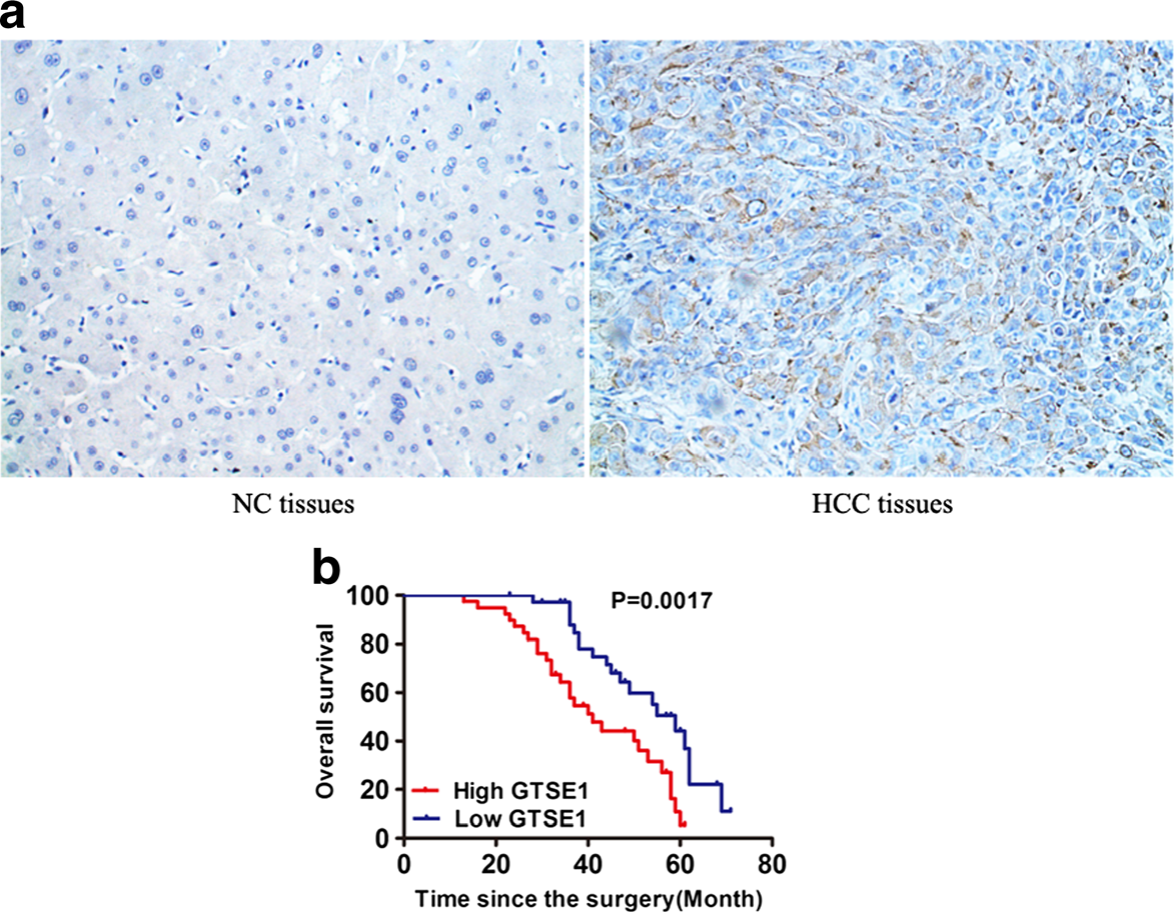
High GTSE1 expression was associated with poor prognosis in HCC. **a** Immunohistochemistry of GTSE1 protein expression in non-cancerous tissues and HCC specimens. GTSE1-negative staining in normal tissue is shown in the *left*, whereas strong GTSE1-positive staining in HCC is presented in the *right*. **b** Kaplan–Meier survival curve was performed to reveal that patients with high GTSE1 expression suffered the worse outcome

To further confirm GTSE1 expression, immunohistochemistry was performed in HCC tissues and paraneoplastic non-cancerous tissues. GTSE1 staining was mainly observed in the cytoplasm of the cells as shown in Fig. 2a. A majority (56/76, 73.7 %) of HCC samples were found to be positive for GTSE1. In contrast, only 22.4 % (17/76) non-cancerous samples were positive for GTSE1. The difference between tumor and non-cancerous specimens was highly significant (*P* < 0.001). The relationship between clinicopathological parameters and GTSE1 expression is summarized in Table 1. High GTSE1 expression positively associated with clinicopathological parameters like tumor size (*P* = 0.0053), venous invasion (*P* = 0.0115), and tumor grade (*P* = 0.0203), whereas other clinic-pathological characteristics have been shown no correlation. In addition, the Kaplan-Meier survival curves were performed to determine the correlation between GTSE1 expression and HCC patient survival. We found a significantly shorter overall survival time (41 months) in patients with higher GTSE1 expression than those with lower GTSE1 level (59 months), as shown in Fig. 2b. Univariate analysis showed that GTSE1, tumor size, vascular invasion, and tumor-node-metastasis stage were significantly associated with OS in HCC patients (Table 2). In addition, multivariate analysis showed that GTSE1 was an independent prognostic indicator for OS (Table 3).

**Table 1:** Associations between GTSE1 protein expression levels and clinicopathologic variables of HCC patients

Clinicopathologic variables	All cases (*n* = 76)	GTSE1 expression		*P* value
		High (39)	Low (37)	
Gender				
Male	67	34	33	1.00
Female	9	5	4	
Age, years				
≤60	44	24	20	0.6426
>60	32	15	17	
Liver cirrhosis				
Absent	42	23	19	0.6448
Present	34	16	18	
Tumor encapsulation				
Absent	36	22	14	0.1222
Present	48	20	28	
Tumor size (cm)				
≤5	34	11	23	0.0053
>5	42	28	14	
Tumor multiplicity				
Single	53	26	27	0.6220
Multiple	23	13	10	
Venous invasion				
Absent	44	17	27	0.0115
Present	32	22	10	
TNM stage				
I–II	41	16	25	0.0203
III	35	23	12	

*P* values were calculated by Fisher’s exact test

*TNM* tumor-node-metastasis

**Table 2:** Univariate analyses of factors associated with overall survival

Variable	Overall survival	
	Hazard ratio (95 % CI)	*P* value
Gender (male vs. female)	2.046 (0.632–6.621)	0.232
Age, years (>52 vs. ≤52)	1.106 (0.613–1.996)	0.738
Liver cirrhosis (yes vs. no)	1.231 (0.685–2.213)	0.488
Tumor encapsulation (none vs. complete)	1.881 (1.019–3.474)	0.043
Tumor size (cm; >5 vs. ≤5)	2.604 (1.338–5.065)	0.005
Tumor number (multiple vs. single)	2.040 (1.134–4.026)	0.057
Vascular invasion (yes vs. no)	2.043 (1.423–3.883)	0.017
TNM stage (II/III vs. I)	3.236 (1.636–6.403)	0.001
GTSE1 (high vs. low)	2.609 (1.394–4.883)	0.003

Univariate analysis, Cox proportional hazard regression model

*95 % CI* 95 % confidence interval, *TNM* tumor-node-metastasis

**Table 3:** Multivariate analyses of factors associated with overall survival

Variable	Overall survival	
	Hazard ratio (95 % CI)	*P* value
GTSE1 (high vs. low)	2.388 (1.193–4.783)	0.014
Tumor encapsulation (none vs. complete)	1.641 (0.716–3.757)	0.242
Tumor size (cm; >5 vs. ≤5)	1.480 (0.607–3.607)	0.389
Vascular invasion (yes vs. no)	1.066 (0.448–2.533)	0.885

Mutivariate analysis, Cox proportional hazards regression model

*95 % CI* 95 % confidence interval

### GTSE1 knockdown suppresses tumor cell proliferation, arrested cell cycle, and induced cell apoptosis

**Fig. 3:**
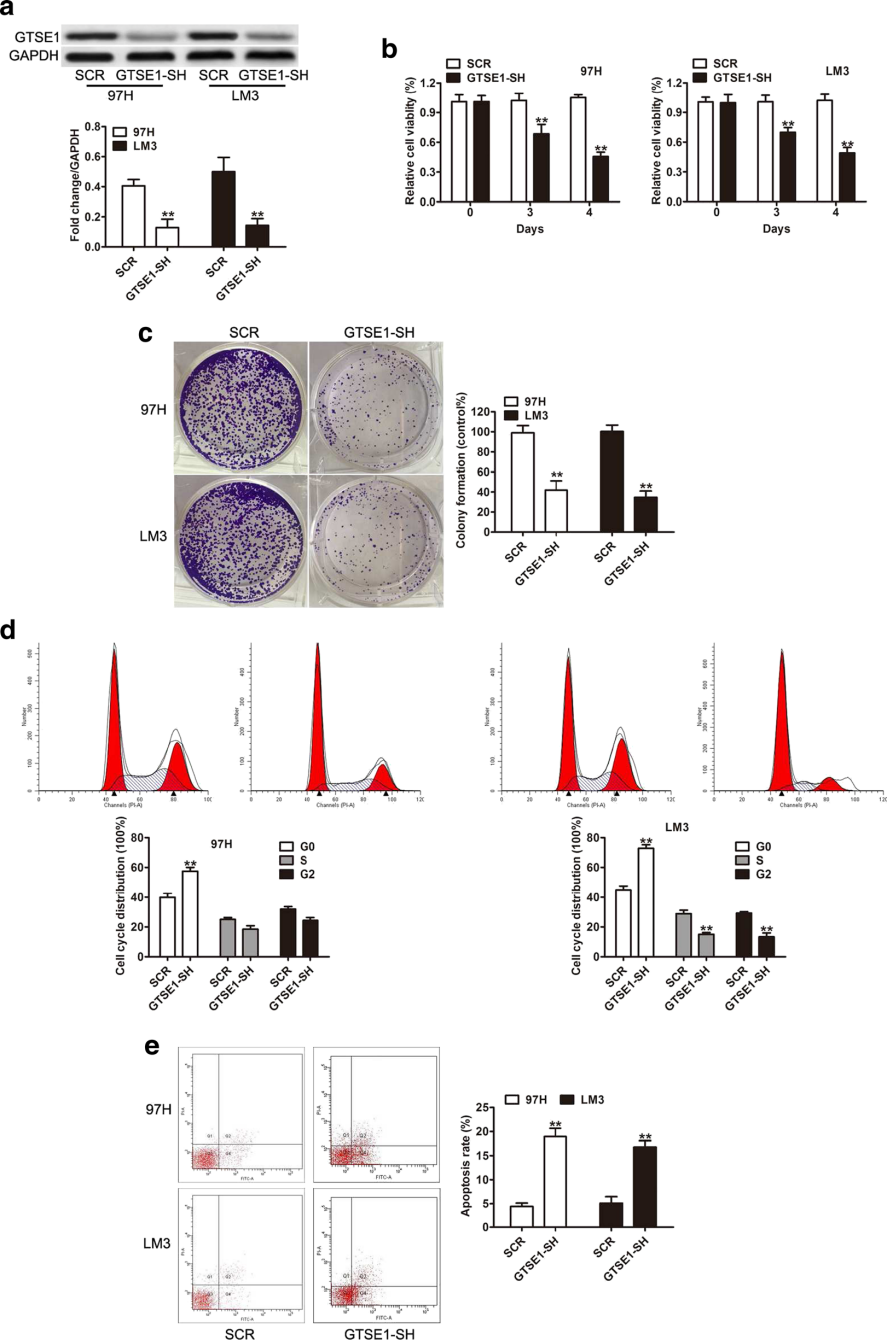
Silencing of GTSE1 inhibited HCC cell growth. **a** Western blots were performed to confirm GTSE1 stably downregulated in 97H and LM3 cells. **b** The CCK-8 assay was used to quantify the relative cell viability at indicated time points. **c** Representative pictures of colony formation assay in 97H and LM3 cells transfected with or without GTSE1. **d** The ratio of cells at different cell cycle phases was evaluated by flow cytometric analysis and quantitative analysis of the different cell cycle phases. **e** Cell apoptosis of 97H and LM3 cells transfected with SCR or GTSE1-SH was assessed by flow cytometric analysis. **P < 0.01

Since GTSE1 overexpression was observed in HCC tissues and cells, our next question is whether GTSE1 had a direct functional role in facilitating tumor growth in HCC. Stable knockdown of GTSE1 in 97H and LM3 cells was constructed via lentiviral infection by and confirmed by western blotting analysis (Fig. 3a). Cell proliferation assay indicated that GTSE1 silencing significantly inhibited cell proliferation both in 97H and LM3 cells (*P* < 0.01, Fig. 3b). Colony formation assay also suggested that GTSE1 knockdown significantly reduced the number and size of cell colonies formed compared with the SCR group (Fig. 3c). Furthermore, flow cytometric analysis was performed to evaluate whether the effect of GTSE1 on proliferation of HCC cells affected cell-cycle progression and apoptosis. Our data showed that downregulation of GTSE1 expression leads to a significant increase of G0/G1 phase compared with negative control (*P* < 0.01, Fig. 3d). Apoptotic assay also showed that knockdown GTSE1 could obviously promote cell apoptosis (*P* < 0.01, Fig. 3e). These findings indicated that GTSE1 might play as an oncogene in HCC.

### GTSE1 knockdown inhibited cell migration and invasion

**Fig. 4:**
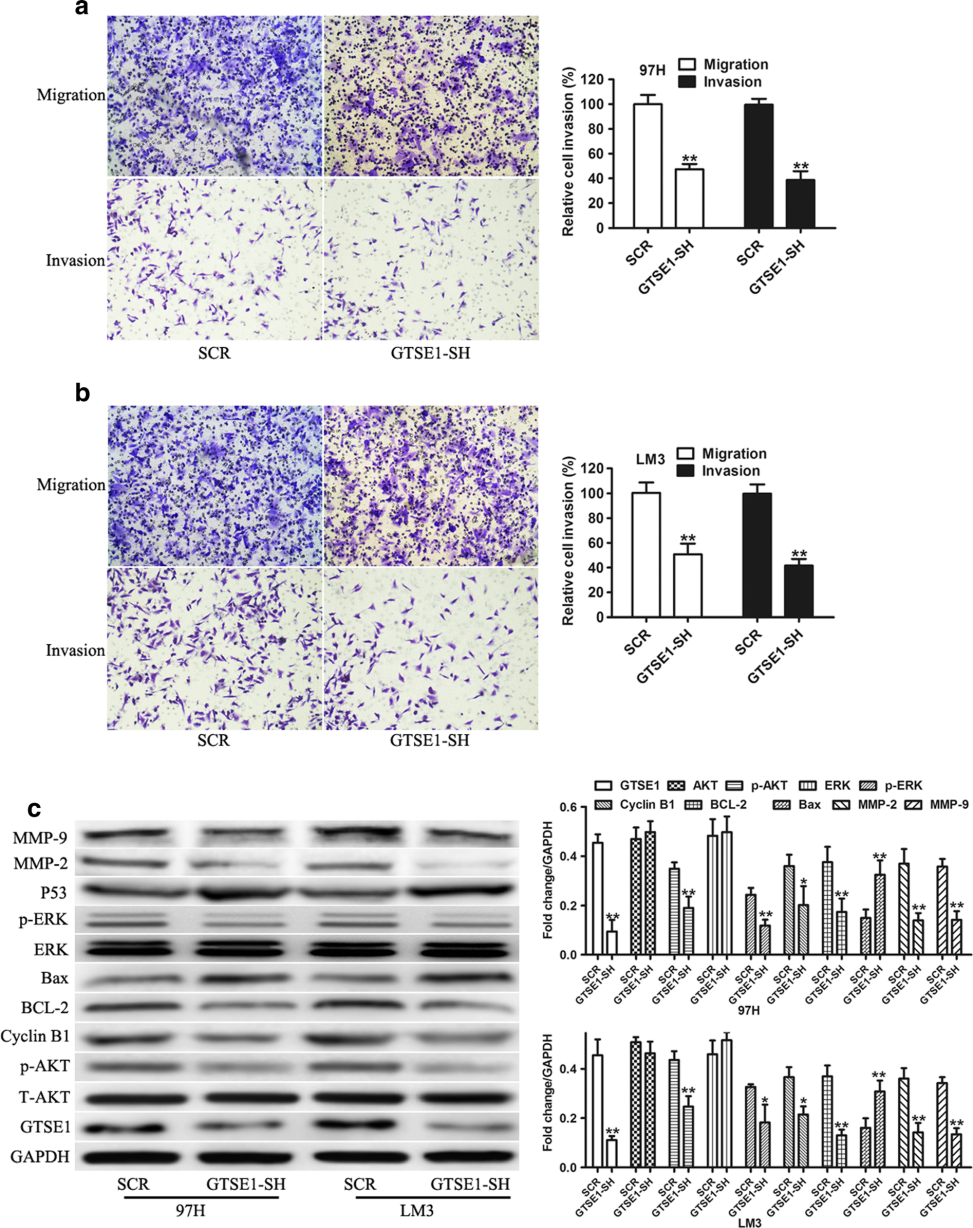
GTSE1 knockdown suppressed cell invasion and regulated AKT phosphorylation. **a** Matrigel-uncoated/coated transwell cell invasion assays of 97H cells transfected with SCR or GTSE1-SH. **b** Matrigel-uncoated/coated transwell cell invasion assays of LM3 cells transfected with SCR or GTSE1-SH. **c** Western blot detection of GTSE1, ATK, p-AKT, ERK, p-ERK, BCL-2, Bax, cyclin B1, p53, MMP-2, and MMP-9 expression in 97H and LM3 cells transfected with SCR or GTSE1-SH. **P < 0.01

As clinical data shown, high GTSE1 expression was associated with venous invasion. Thus, GTSE1 may play an important role in HCC cell migration and invasion that is very important for tumor metastasis. Transwell assays were used to explore the effect of GTSE1 on the motile and invasive phenotype of HCC cells. Migration and invasion were significantly reduced in GTSE1 knock downed 97H cells compared with control cells (*P* < 0.01, Fig. 4a). The same results were also observed by using another HCC cell line, LM3 (*P* < 0.01, Fig. 4b).

Next, we sought to determine how GTSE1 exerted these biological effects by characterizing its effect on known functional molecule and signaling pathways. Accumulated reports have shown that the PI3K/AKT and ERK/MAPK are the signaling pathways for tumor growth and invasion (Mishra et al. 2012; Sui et al. 2015). Therefore, we assessed the effect of GTSE1 on the levels of phosphorylation of AKT and ERK. Our results showed that GTSE1 silencing resulted in a decrease of phosphorylation of AKT and ERK and a downstream target BCL-2, MMP-2, and MMP-9, an increase of Bax and p53 both in 97H and LM3 cells (Fig. 4c). Compared with negative control cells, GTSE1 downregulation in 97H and LM3 cells decreased cyclin B1 protein expression (Fig. 4c), a crucial regulatory protein in G2/M phase. These findings suggested that GTSE1 silencing inhibits the proliferation and invasion of HCC cells may be mediated by affecting the phosphorylation level of AKT and the expression of BCL-2, Bax, and cyclin B1.

### Knockdown of GTSE1 suppresses tumor growth in vivo

**Fig. 5:**
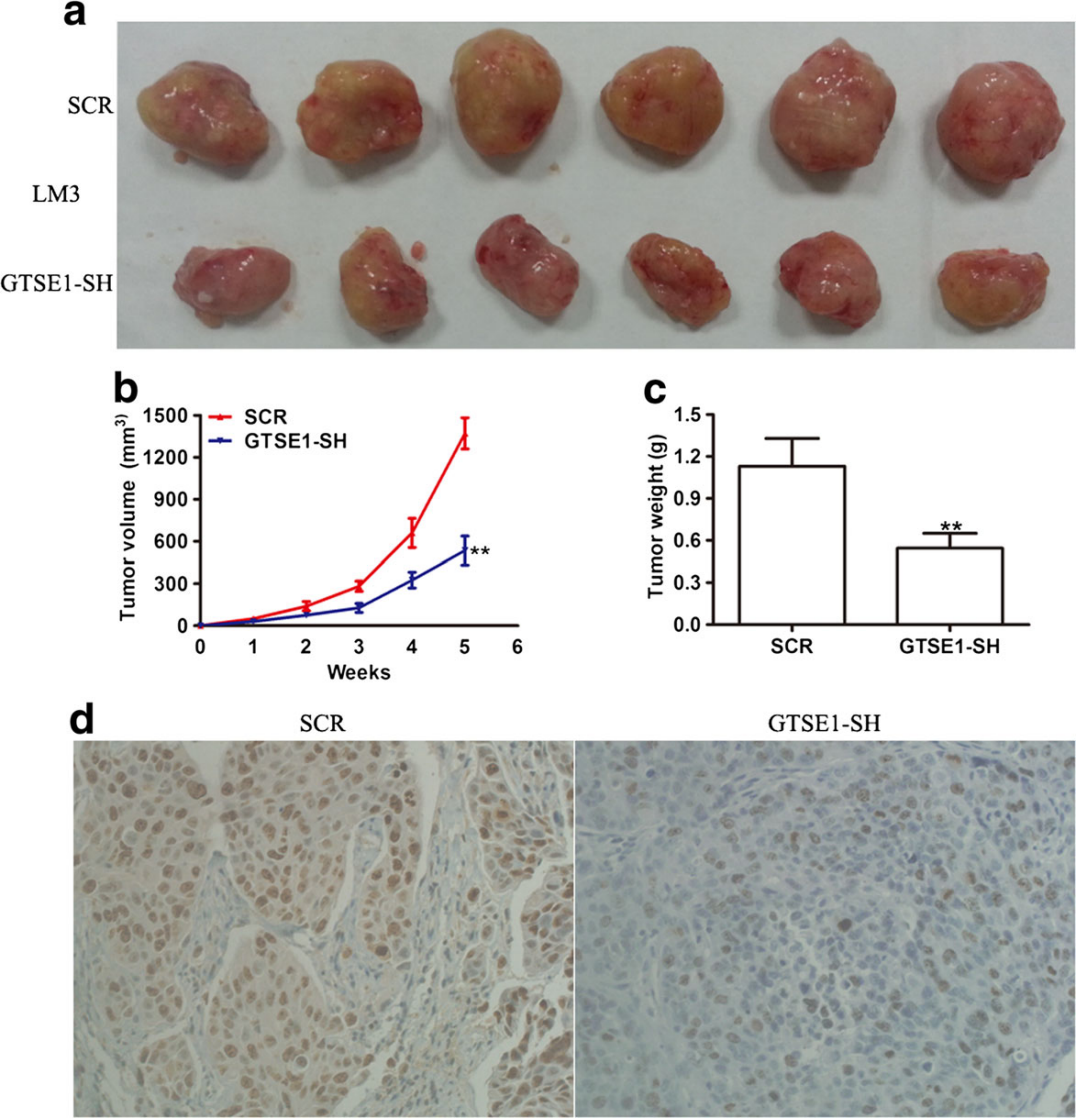
Knockdown of GTSE1 inhibits the growth of HCC in vivo. **a** Representative pictures of subcutaneous implantation tumor of LM3 cellsat 5 weeks after injection were shown. **b** Tumor growth curves of subcutaneous implantation models of LM3 cells at indicated time points. **c** Tumor weights were quantified. Data were represented as the mean ± SD of three independent experiments. **d** Representative images of Ki67 in tumor xenograft tissues from subcutaneously inoculated Nu/Nu mice (original magnifications: ×400). **P < 0.01

Given that GTSE1 silencing impaired the growth of HCC cells in vitro, we determined whether GTSE1 silencing could affect tumorigenicity in vivo. We injected LM3 cells transfected with either SCR or GTSE1silencing into nude mice. Consistent with results in vitro, tumor growth in GTSE1 silencing group was obviously slower than that in the SCR group (Fig. 5a, b). The average tumor weight in GTSE1 silencing group was markedly lower than that in the SCR group (Fig. 5c). In addition, transplantation tumor specimens were resected and stained with Ki67 by immunohistochemistry. The result showed that the expression of Ki67 was reduced in the GTSE1 knockdown group, which revealed a low cell proliferation compared with the SCR group in vitro (Fig. 5d). These data support a role for GTSE1 upregulation in promoting the formation and growth of tumor xenografts in vivo.

## Discussion

The abnormal expression of genes has been reported in many types of cancer, and a number of genes have been explored in the process of cancer development (Zhu et al. 2013; Cao et al. 2014). In our present study, we first found that GTSE1 was frequently upregulated in HCC tissues and cells and the increased GTSE1 expression was closely related to tumor size, venous invasion, and advanced stage of HCC. Furthermore, loss of function assays on GTSE1 indicated that inhibition of GTSE1 was not only significantly decreased HCC tumor growth in vitro and in vivo but also suppressed the migration and invasion in vitro. Our results showed that GTSE1 might play a proto-oncogenic role in HCC development and may be useful as a therapeutic target for HCC.

GTSE1, a negative regulator of p53, was a microtubule-localized protein, and its upregulation contributes to the aggressive behavior of several cancer types (Tian et al. 2011; Spanswick et al. 2012; Subhash et al. 2015; D’Errico et al. 2009). Higher GTSE1 expression is associated with more aggressive phenotypes in neuroendocrine tumors (Lee et al. 2012). Consistent with these results, our results demonstrated that GTSE1 was upregulated in HCC tissues and associated with tumor size, venous invasion, advanced stage, and patient overall survival. These data strongly indicated that GTSE1 could play a crucial role in HCC progression. Furthermore, our current data also showed that GTSE1 knockdown inhibited proliferation, migration, and invasion and induced apoptosis of HCC cells in vitro, which is consistent with the data on breast cancer (Scolz et al. 2012). Previous studies have also reported that GTSE1 may play a role in the regulation of cell cycle via promoting p53 localization to the cytoplasm, which is influencing cell cycle distribution (Monte et al. 2003; Liu et al. 2010). In both 97H and LM3 cells, knockdown of GTSE1 resulted in an increase of G0/G1 phase and a decrease of G2/M phase. Then, the expression of cyclin b1 and p53 also indicated the alteration of GTSE1 that regulated the distribution of cell cycle.

In order to understand the contribution of GTSE1 to HCC progress, it would be of great interest to identify signaling cascades by which GTSE1 promoted proliferation and invasion. Previous studies showing that AKT and ERK were associated with survival pathway and apoptosis pathway (Bratton et al. 2010; Ding et al. 2015), we then inquired about whether GTSE1 utilized the same pathway to affect cell growth and invasion. In this study, knockdown of GTSE1 downregulated phosphorylation of AKT, BCL-2, and cyclin B1 but upregulated Bax, which suggested that GTSE1 contributes to high aggressive behavior of HCC through its enhancing effect on AKT survival pathway and apoptosis pathway.

In summary, our study demonstrated that GTSE1 was overexpressed in HCC at both mRNA and protein levels and a poor prognostic marker. GTSE1 knockdown was found to affect cell proliferation, migration, and invasion of HCC cells through dysregulation of AKT, BCL-2, Bax, and cyclin B1. Further investigation of GTSE1 might provide potentially useful information for developing biological or pharmacological agents for HCC patients.

## Electronic supplementary material

Below is the link to the electronic supplementary material.

ESM 1 (XLS 32 kb)

## References

[CR1] Bratton MR, Duong BN, Elliott S, Weldon CB, Beckman BS, McLachlan JA (2010). Regulation of ERalpha-mediated transcription of Bcl-2 by PI3K-AKT crosstalk: implications for breast cancer cell survival. Int J Oncol.

[CR2] Cao Z, Fu B, Deng B, Zeng Y, Wan X, Qu L (2014). Overexpression of Chemokine (C-X-C) ligand 1 (CXCL1) associated with tumor progression and poor prognosis in hepatocellular carcinoma. Cancer Cell Int.

[CR3] Cavazza A, Caballeria L, Floreani A, Farinati F, Bruguera M, Caroli D (2009). Incidence, risk factors, and survival of hepatocellular carcinoma in primary biliary cirrhosis: comparative analysis from two centers. Hepatology.

[CR4] D’Errico M, de Rinaldis E, Blasi MF, Viti V, Falchetti M, Calcagnile A (2009). Genome-wide expression profile of sporadic gastric cancers with microsatellite instability. Eur J Cancer.

[CR5] Ding J, Li QY, Yu JZ, Wang X, Lu CZ, Ma CG (2015). The lack of CD131 and the inhibition of Neuro-2a growth by carbamylated erythropoietin. Cell Biol Toxicol.

[CR6] Dong Q, Zhu X, Dai C, Zhang X, Gao X, Wei J, et al. Osteopontin promotes epithelial-mesenchymal transition of hepatocellular carcinoma through regulating vimentin. Oncotarget. 2016. 10.18632/oncotarget.7016 PMC4914337 26824421

[CR7] Lee J, Sung CO, Lee EJ, Do IG, Kim HC, Yoon SH (2012). Metastasis of neuroendocrine tumors are characterized by increased cell proliferation and reduced expression of the ATM gene. PLoS ONE.

[CR8] Liu XS, Li H, Song B, Liu X (2010). Polo-like kinase 1 phosphorylation of G2 and S-phase-expressed 1 protein is essential for p53 inactivation during G2 checkpoint recovery. EMBO Rep.

[CR9] Liu Y, Zhang JB, Qin Y, Wang W, Wei L, Teng Y (2013). PROX1 promotes hepatocellular carcinoma metastasis by way of up-regulating hypoxia-inducible factor 1alpha expression and protein stability. Hepatology.

[CR10] Liu Y, Flynn TJ, Xia M, Wiesenfeld PL, Ferguson MS (2015). Evaluation of CYP3A4 inhibition and hepatotoxicity using DMSO-treated human hepatoma HuH-7 cells. Cell Biol Toxicol.

[CR11] Mishra V, Ansari KM, Khanna R, Das M (2012). Role of ErbB2 mediated AKT and MAPK pathway in gall bladder cell proliferation induced by argemone oil and butter yellow. Argemone oil and butter yellow induced gall bladder cell proliferation. Cell Biol Toxicol.

[CR12] Monte M, Collavin L, Lazarevic D, Utrera R, Dragani TA, Schneider C (2000). Cloning, chromosome mapping and functional characterization of a human homologue of murine gtse-1 (B99) gene. GENE.

[CR13] Monte M, Benetti R, Buscemi G, Sandy P, Del SG, Schneider C (2003). The cell cycle-regulated protein human GTSE-1 controls DNA damage-induced apoptosis by affecting p53 function. J Biol Chem.

[CR14] Monte M, Benetti R, Collavin L, Marchionni L, Del SG, Schneider C (2004). HGTSE-1 expression stimulates cytoplasmic localization of p53. J Biol Chem.

[CR15] Poon RT (2011). Prevention of recurrence after resection of hepatocellular carcinoma: a daunting challenge. Hepatology.

[CR16] Scolz M, Widlund PO, Piazza S, Bublik DR, Reber S, Peche LY (2012). GTSE1 is a microtubule plus-end tracking protein that regulates EB1-dependent cell migration. PLoS ONE.

[CR17] Spanswick VJ, Lowe HL, Newton C, Bingham JP, Bagnobianchi A, Kiakos K (2012). Evidence for different mechanisms of ‘unhooking’ for melphalan and cisplatin-induced DNA interstrand cross-links in vitro and in clinical acquired resistant tumour samples. BMC Cancer.

[CR18] Subhash VV, Tan SH, Tan WL, Yeo MS, Xie C, Wong FY (2015). GTSE1 expression represses apoptotic signaling and confers cisplatin resistance in gastric cancer cells. BMC Cancer.

[CR19] Sui Y, Zheng X, Zhao D. Rab31 promoted hepatocellular carcinoma (HCC) progression via inhibition of cell apoptosis induced by PI3K/AKT/Bcl-2/BAX pathway. Tumour Biol. 2015. 10.1007/s13277-015-3626-5 26044564

[CR20] Tian T, Zhang E, Fei F, Li X, Guo X, Liu B (2011). Up-regulation of GTSE1 lacks a relationship with clinical data in lung cancer. Asian Pac J Cancer Prev.

[CR21] Utrera R, Collavin L, Lazarevic D, Delia D, Schneider C (1998). A novel p53-inducible gene coding for a microtubule-localized protein with G2-phase-specific expression. EMBO J.

[CR22] Wong HC, Wong CC, Sagineedu SR, Loke SC, Lajis NH, Stanslas J (2014). SRJ23, a new semisynthetic andrographolide derivative: in vitro growth inhibition and mechanisms of cell cycle arrest and apoptosis in prostate cancer cells. Cell Biol Toxicol.

[CR23] Zhu WW, Guo JJ, Guo L, Jia HL, Zhu M, Zhang JB (2013). Evaluation of midkine as a diagnostic serum biomarker in hepatocellular carcinoma. Clin Cancer Res.

[CR24] Zhu X, Li D, Yu F, Jia C, Xie J, Ma Y, et al. MiR-194 inhibits the proliferation, invasion, migration, and enhances the chemosensitivity of non-small cell lung cancer cells by targeting forkhead box A1 protein. Oncotarget. 2016. 10.18632/oncotarget.7545 PMC4914347 26909612

